# Epidemiological and clinical features of children with the Omicron BA.5.2 subvariant in Guangzhou

**DOI:** 10.1038/s41392-023-01372-0

**Published:** 2023-04-26

**Authors:** Feng Li, Yinghua He, Xianglong Lan, Lu Li, Fei Gu, Zhao Ye, Lv Wang, Zhenghui Cen, Weichun Zhu, Haisheng Yu

**Affiliations:** Guangzhou Eighth People’s Hospital, Guangzhou Medical University, Guangzhou, 510440 China

**Keywords:** Infectious diseases, Immunological disorders, Infectious diseases

**Dear Editor**,

Coronavirus disease 2019 (COVID-19) is a novel infectious disease caused by severe acute respiratory syndrome coronavirus 2 (SARS-CoV-2). Compared with previous variants, Omicron strains are more easily transmissible, have a greater ability to evade the human immune response, and are characterized as naturally virulence attenuated.^1^ During the early stages of the COVID-19 pandemic, it became apparent that children were less easily infected than adults. However, the current Omicron pandemic in China has seen a high rate of infection among children and adolescents. Information on the epidemiological and clinical characteristics of the Omicron BA.5.2 subvariant in children is limited. In this study, we aimed to investigate the clinical characteristics of Omicron BA.5.2 in children and adolescents.

Data were gathered from the medical records of Guangzhou Eighth People’s Hospital during October 1st, 2022 to November 30th, 2022 on patients aged ≤18 years diagnosed with Omicron BA.5.2 variant. We identified 683 such cases. These were categorized into four groups according to the age of onset: 0–3 years (0–3 y, *n* = 265), 4–6 years (4–6 y, *n* = 134), 7–14 years (7–14 y, *n* = 212), and 15–18 years (15–18 y, *n* = 72) (Fig. 1a). Of these patients, 390 (57.1%) had previously received two or three doses of inactivated SARS-CoV-2 vaccine. The vaccination rates among the cohort were 84.33% (103/134) in the 4–6 y group, 95.28% (202/212) in the 7–14 y group, and 93.06% (67/72) in the 15–18 y group respectively. In the 0–3 y group, 97% had received no vaccinations against SARS-CoV-2 (Fig. 1b and Supplementary Table 1). This is because the official policy in China stipulates that children under 3 years are not eligible for SARS-CoV-2 vaccination.

**Fig. 1:**
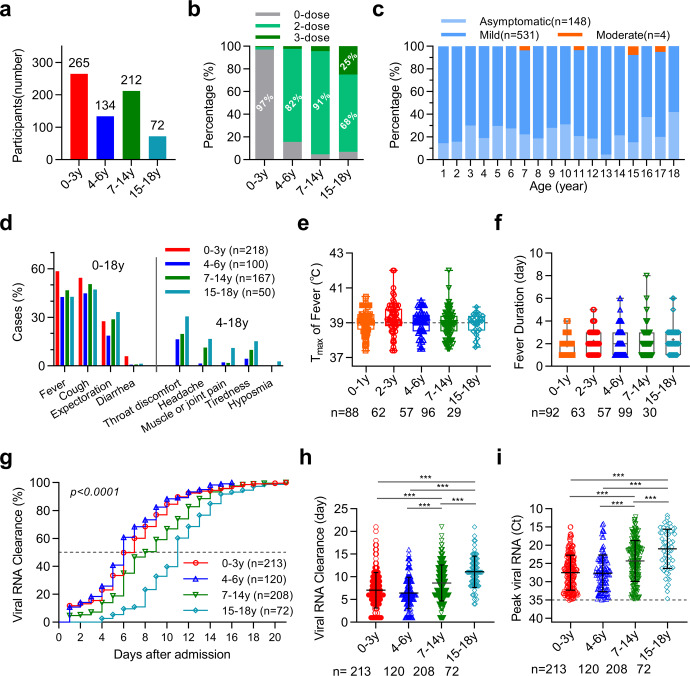
Clinical characteristics of patients aged 0–18 with the SARS-CoV-2 Omicron subvariant BA.5.2 in Guangzhou. **a** The number of pediatric and adolescent patients with the BA.5.2 strain of COVID-19 in four age groups. Patient numbers are shown above each group. **b** The percentages of patients in four age groups who have received a SARS-CoV-2 vaccine. **c** The percentages of asymptomatic, mild, and moderate cases of the BA.5.2 strain of COVID-19 in patients aged 0–18 years. **d** The rates of fever, cough, expectoration, and diarrhea in patients aged 0–18 years and the rates of throat discomfort, headache, muscle or joint pain, tiredness, and hyposmia in patients with BA.5.2 in patients aged 4–18 years. **e**, **f** Fever peaks and fever duration (in days) in BA.5.2 patients of four age groups. Patient numbers are shown below each group. **g** The kinetics of SARS-CoV-2 RNA clearance. Cumulative viral clearance (%) is shown. The abscissa represents the time required for negative conversion of nucleic acid and the ordinate represents the percentages of patients with negative nucleic acid. The calculation method for viral RNA clearance was: viral RNA clearance (%) = the number of patients whose nucleic acid turned negative/total number of patients × 100%. *P*-values (derived by log-rank [Mantel-Cox] test) are shown. **h** Time for viral clearance. **i**. Peak viral load. The maximum viral load (represented by the lowest Ct value) of each patient during hospitalization was identified. Patient numbers are shown below each group. Each dot represents one patient. The Mann–Whitney U-test was used for (**e**), (**f**), (**h**) and (**i**); *p*-values <0.05 were deemed statistically significant (*p < 0.05, **p < 0.01, ***p < 0.001). 0–3 y, patients aged 0–3 years old; 4–6 y, patients aged 4–6 years old; 7–14 y, patients aged 7–14 years old; 15–18 y, patients aged 15–18 years old. 0-dose, not vaccinated; 2-dose, received two doses of the vaccine; 3-dose, received three doses of the vaccine. Ct, cycle threshold; RNA, ribonucleic acid; SARS-CoV-2, severe acute respiratory syndrome coronavirus 2

We next compared the clinical characteristics of the four groups. In all four groups, the majority of cases showed mild symptoms (82.26%, 74.63%, 77.83 %, and 66.67%, from youngest to oldest age group) followed by asymptomatic cases (17.74%, 25.37%, 21.23%, and 30.55%, from youngest to oldest group) (Supplementary Table 1). In all 683 cases, there were only four moderate cases. These were seen in the 7–14 y and the 15–18 y groups (Fig. 1c). There were no severe or critical cases. Of the 535 symptomatic cases, the common clinical symptoms were fever (63.74%), cough (64.49%), expectoration (34.21%) and diarrhea (3.74%) (Supplementary Table 1). The three groups covering ages 4–18 accounted for 317 of the symptomatic cases. Among these, 86 (27.13%) patients complained of throat discomfort, 38 (11.99%) of headache, 15 (4.73%) of muscle or joint pain, 38 (11.99%) of tiredness and 2 (0.63%) of hyposmia (decreased sense of smell) (Supplementary Table 1). Fever is known to be a predominant symptom in all variants of COVID-19 and is a common immune response. This was seen with particular frequency in the 0–3 y group (Fig. 1d). The peak fever of most of the infants aged 0–1 year was over 39 °C. This was higher than that seen in the other age groups, but the duration of fever was shorter, on average, in 0–1 year group (Fig. 1e, f). However, compared with the other three groups combined (4–18 y), the mean of fever peak and the mean of fever duration in children aged 2–3 years were not significantly different.

We further analyzed the dynamic changes in the viral load in all cases that were estimated with cycle threshold (Ct) values by RT-PCR. In all four age groups, the highest viral loads were recorded on the day of hospital admission, with viral loads decreasing over time (Supplementary Fig. 1). Among the four groups, the viral clearance time was fastest in the 4–6 y group. The 0–3 y group cleared viral ribonucleic acid (RNA) faster than the older age groups (Fig. 1g and h). As shown in Fig. 1i, the viral load was lowest in the youngest group and increased progressively with age, being highest in the oldest group. The viral load in children aged 0–14 years was significantly lower than that in adolescents aged 15–18 years.

We also analyzed the immunoglobulin M (IgM) levels of our cohort (Fig. S2A) and found that almost all of the BA.5.2 patients were IgM-negative during the first three days after admission (Supplementary Fig. 2a). On the seventh day after admission, IgM levels remained low in all four age groups (Supplementary Fig. 2b). Further, these laboratory results were analyzed for all patients on the day of hospital admission. Compared with children aged 0–3 years, markly decreased monocytes counts and significantly increased hemoglobin level were observed in the three age groups. Compared with children aged 0–6 years, leukocytes counts and lymphocytes counts significantly decreased, and hemoglobin level significantly increased in patients aged 7–14 years and 15–18 years (Supplementary Table 2).

In this study, we summarized the clinical characteristic of children and adolescents infected with omicron BA.5.2 among the different age groups. Most of the patients were asymptomatic or mild (over 99%), a few were moderate, and no severe or critical case was observed. The infants aged 0–1 year had higher fever peaks, but frequently experienced fever with a short duration. Additionally, compared with adolescents, we found that viral loads were lower and viral clearance time was significantly shorter in children, especially in the 4–6 y group.

Our study found that the viral loads of our cohort were significantly lower in the three younger groups (0–14 y) combined than in the adolescent group (15–18 y). The time to SARS-CoV-2 RNA-negative conversion was also significantly shorter in the three child groups combined than in the adolescent group. Such differences between children and adolescents have received some previous research attention in immunological studies. Firstly, pre-activated antiviral innate immunity in the upper respiratory tract of children can control SARS-CoV-2 infection in early childhood with a stronger antiviral response than in adults.^2^ Secondly, children convalescing from COVID-19 show more robust humoral immune responses to SARS-CoV-2 than convalescent adults.^3^ Additionally, ACE2 gene expression is significantly lower in younger than older children. As ACE2 is the protein to which SARs-CoV-2 binds, this is probably the reason for the difference in viral loads between age groups.^4^

This study had several limitations. Firstly, this was a retrospective, observational study only, and the data gathered pertained to the clinical symptoms and treatment strategies of children with SARS-CoV-2, especially those aged 0–3 years. Secondly, the clinical data were gathered from a single institution in Guangzhou, China (Guangzhou Eighth People’s Hospital), and this limits the generalization of our findings. Thirdly, there is, as yet, no quantitative test for virus-specific antibodies such as immunoglobulin A and immunoglobulin G in children and adolescents. Fourthly, although the viral sub-lineage and critical mutations are important prognostic indicators in children, it is too hard to sequence the virus strain in every infected child under China’s COVID-19 emergency policy at that time.

## Supplementary information

Supplementary_data

Dataset 1-7

## Data Availability

The online version of this article contains supplementary material, which is available to authorized users.
