# Supplementary material for: Epidemiological and clinical features of children with the Omicron BA.5.2 subvariant in Guangzhou
Source: Signal Transduct Target Ther. 2023 Apr 26;8:163. doi: 10.1038/s41392-023-01372-0 (PMC10130145; doi:10.1038/s41392-023-01372-0)
Supplement: Supplementary file 1 — Supplementary_data [file 41392_2023_1372_MOESM1_ESM.docx]

Supplementary Materials for

**Epidemiological and clinical features of children with the Omicron BA.5.2 subvariant in Guangzhou**

Feng Li ^#^, Yinghua He^#^, Xianglong Lan, ^#^ Lu Li, Fei Gu, Zhao Ye, Lv Wang, Zhenghui Cen, Weichun Zhu^*^, Haisheng Yu ^*^.

Correspondence to: yuhaisheng@gzhmu.edu.cn

**This file includes:**

Materials and Methods

Supplementary Figure1 to 2

Supplementary Tables1 to 2

**Patient Information and Data Collection**

All of the patients in this study were treated at Guangzhou Eighth People’s Hospital, Guangzhou Medical University between October 1st and November 30th, 2022. During this period, 683 patients aged ≤18 years were diagnosed with the Omicron BA.5.2 variant of SARS-CoV-2 by sequencing or epidemiologic investigation. The nasopharyngeal swabs collected from children or parents of children in our cohort were spot-checked for analysis by whole genome sequencing of SARS-CoV-2 viral RNA. The sequencing results showed the patients to be infected with the Omicron BA.5.2 strain.

Based on schooling stages and vaccine eligibility, the children and adolescents were divided into four age groups: 0–3 years (young children), 4–6 years (preschool age), 7–14 years (school age), and 15–18 years (adolescents) ^1-6^. The general treatment strategy for symptomatic children was as follows. 1) Reduce fever and prevent convulsions. This was achieved using antipyretics such as acetaminophen or ibuprofen and anticonvulsants such as midazolam, diazepam, or phenobarbital. 2) Relieve coughing and phlegm. This was achieved through the oral administration of ambroterol. 3) Reduce allergenic symptoms. This was achieved using loratadine or cetirizine. The laboratory results and demographic and clinical characteristics of the patients in our sample were collected from the hospital information system and laboratory information system. This study was approved by the Guangzhou Eighth People’s Hospital Ethics Committee (202209221). The participant (or parent/guardian if the participant is a minor or incapable or legal representative) gave written informed consent for publication. The research was conducted in strict accordance with the rules and regulations of the Chinese government for the protection of human research participants.

**COVID-19 Disease Severity Classification**

COVID-19 severity was graded according to the *Diagnosis and Treatment Protocol for COVID-19 (trial version 9)* by the Chinese National Health Commission. Asymptomatic cases were those who tested positive for nucleic acid or SARS-CoV-2 viral RNA but showed no clinical symptoms. Mild cases were those with mild clinical symptoms but no signs of pneumonia on imaging. Moderate cases were those with a fever, respiratory manifestations, and signs of pneumonia on imaging. Severe cases were those who met any of the following criteria: (1) shortness of breath, with a respiratory rate ≥30 breaths/min; (2) resting state oxygen saturation ≤93%; (3) arterial partial pressure of oxygen (PaO2)/ inhaled oxygen concentration (FiO2) ≤300 mmHg (1 mmHg=0.133 kPa); or (4) progressive worsening of clinical symptoms and progression of pneumonia lesions >50% within 24 to 48 hours on imaging. Critical cases were those who met any of the following criteria: (1) respiratory failure requiring mechanical ventilation; (2) shock; (3) failure of a non-respiratory organ requiring ICU monitoring and treatment.

**Viral RNA Detection with RT-PCR**

The detailed protocol for sample collection and viral RNA detection was as previously described^7,8^. Briefly, nasopharyngeal or oropharyngeal swabs were collected according to standardized procedures. Viral RNA extraction and RT-PCR detection were performed using a nucleic acid isolation kit (Da’an Gene Co. Ltd., Guangzhou, Guangdong, China; cat no: DA0630) and an RNA detection kit (Da’an Gene Co. Ltd; cat no: DA0930) according to the manufacturer’s instructions. Results were positive if the Ct value was below 40 (200 copies/ml) either for N or the ORF1a/b gene.

**Statistical Analysis**

The data were analyzed using SPSS software, version 25 (IBM Corp., Armonk, NY, USA). The categorical variables were compared using chi-square or Fisher’s exact test. The continuous variables were compared by log-rank (Mantel-Cox) or Kruskal-Wallis tests with post hoc Bonferroni corrections. Comparisons between two groups were made using Mann–Whitney U tests. A p-value <0.05 was deemed statistically significant.

Reference

1 Acker, K. P. *et al.* Indications for Hospitalization in Children with SARS-CoV-2 Infection during the Omicron Wave in New York City. *Children (Basel).* **9**, 1043 (2022).

2 Bertran, M. *et al.* COVID-19 deaths in children and young people in England, March 2020 to December 2021: An active prospective national surveillance study. *PLoS Med.* **19**, e1004118 (2022).

3 Chung, E. *et al.* Comparison of Symptoms and RNA Levels in Children and Adults With SARS-CoV-2 Infection in the Community Setting. *JAMA Pediatr.* **175**, e212025 (2021).

4 Edward, P. R. *et al.* Severity of Illness Caused by Severe Acute Respiratory Syndrome Coronavirus 2 Variants of Concern in Children: A Single-Center Retrospective Cohort Study. *J Pediatric Infect Dis Soc.* **11**, 440-447 (2022).

5 Marks, K. J. *et al.* Hospitalizations of Children and Adolescents with Laboratory-Confirmed COVID-19 - COVID-NET, 14 States, July 2021-January 2022. *MMWR Morb Mortal Wkly Rep.* **71**, 271-278 (2022).

6 Oeser, C. *et al.* Following the Omicron wave, the majority of children in England have evidence of previous COVID infection. *J Infect.* (2022).

7 Wang, Y. *et al.* Transmission, viral kinetics and clinical characteristics of the emergent SARS-CoV-2 Delta VOC in Guangzhou, China. *EClinicalMedicine.* **40**, 101129 (2021).

8 Hu, F. *et al.* A compromised specific humoral immune response against the SARS-CoV-2 receptor-binding domain is related to viral persistence and periodic shedding in the gastrointestinal tract. *Cell Mol Immunol.* **17**, 1119-1125 (2020).

Supplementary Figure 1.


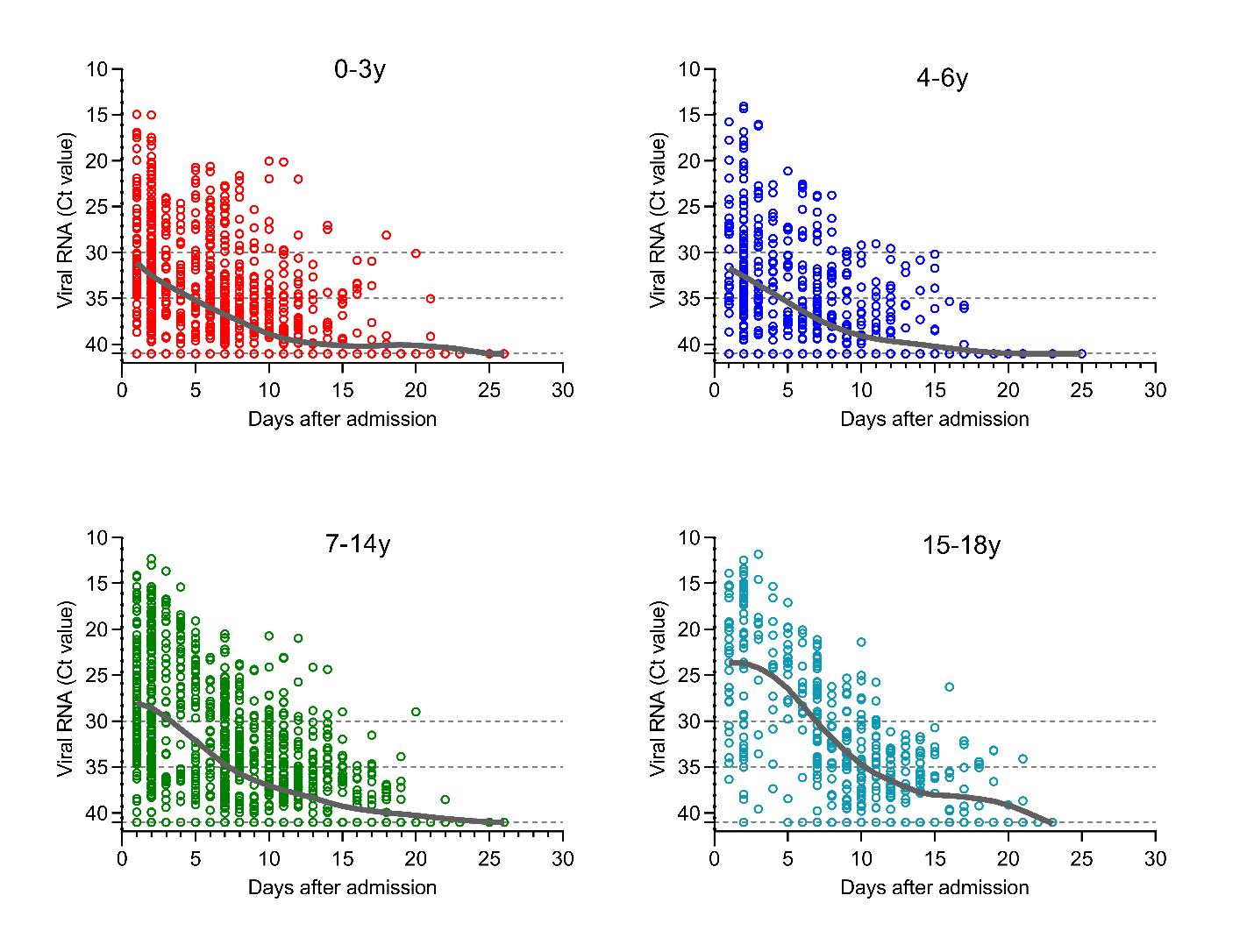
**Supplementary Figure 1.** **The dynamics of viral RNA shedding in the BA.5.2 Omicron subvariant of COVID-19.** Overall changes in viral RNA (Ct value) during hospitalization in the four patient age groups. Each point represents one measurement. Fitted curves for the distribution of the Ct values are shown. Smooth curves and shaded regions indicate 95% CIs. CI, confidence interval; Ct, cycle threshold; RNA, ribonucleic acid.


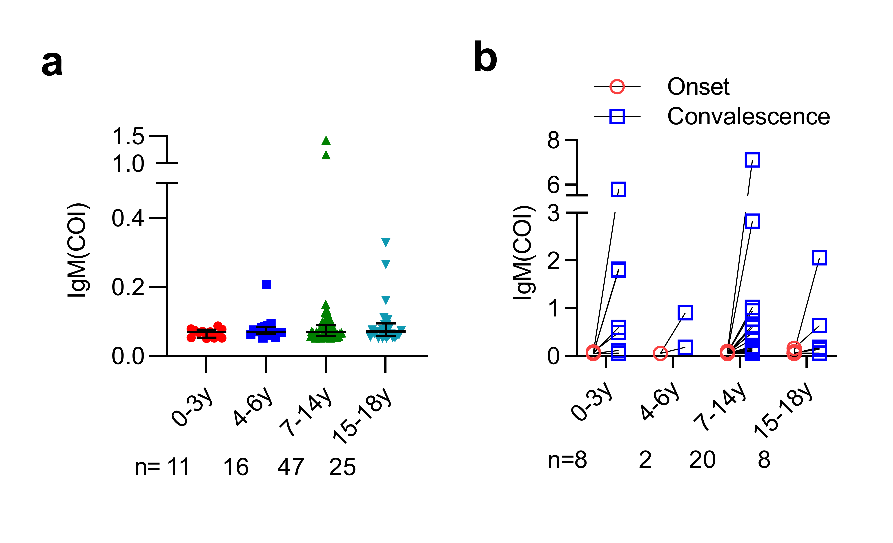
Supplementary Figure 2.

**Supplementary Figure 2.** **Analysis of the SARS-CoV-2 RBD-specific IgM antibody.** **a.** The serum RBD-IgM levels of patients in the four age groups, obtained during the first three days after hospital admission. The IgM (COI) >1 was defined as positive. **b.** The levels of serum IgM of the patients in the four age groups at admission and on the seventh day after admission. COI, cut-off index; IgM, immunoglobulin M; RBD, receptor-binding domain; SARS-CoV-2, severe acute respiratory syndrome coronavirus.

Supplementary Table 1. Clinical characteristics of patients infected with SARS-CoV-2 BA.5.2 variant

| **Characteristics** | **Total (n=683)** | | **0-3y (n=265)** | | **4-6y (n=134)** | | **7-14y (n=212)** | | **15-18y (n=72)** | | ***P* value** |
| --- | --- | --- | --- | --- | --- | --- | --- | --- | --- | --- | --- |
| Gender, n(%) |  |  |  |  |  |  |  |  |  |  |  |
| Male | 366 | (53.59) | 150 | (56.6) | 75 | (55.97) | 109 | (51.42) | 32 | (44.44) | 0.251 |
| Female | 317 | (46.41) | 115 | (43.4) | 59 | (44.03) | 103 | (48.58) | 40 | (55.56) |  |
| Vaccination^★^, n(%) |  |  |  |  |  |  |  |  |  |  |  |
| 0-dose | 293 | (42.9) | 257 | (96.98) | 21 | (15.67) | 10 | (4.72) | 5 | (6.94) |  |
| 2-dose | 358 | (52.42) | 6 | (2.26) | 100 | (82.09) | 193 | (91.04) | 49 | (68.06) | **<0.001** |
| 3-dose | 32 | (4.68) | 2 | (0.76) | 3 | (2.24) | 9 | (4.24) | 18 | (25) |  |
| Classification, n (%) |  |  |  |  |  |  |  |  |  |  |  |
| Asymptomatic | 148 | (21.67) | 47 | (17.74) | 34 | (25.37) | 45 | (21.23) | 22 | (30.55) | 0.059 |
| Mild | 531 | (77.75) | 218 | (82.26) | 100 | (74.63) | 165 | (77.83) | 48 | (66.67) |  |
| Moderate | 4 | (0.58) | 0 | (0) | 0 | (0) | 2 | (0.94) | 2 | (2.78) |  |
| Severe/Critical | 0 | (0) | 0 | (0) | 0 | (0) | 0 | (0) | 0 | (0) |  |
| Comorbidity, n(%) |  |  |  |  |  |  |  |  |  |  |  |
| No | 677 | (99.12) | 264 | (99.62) | 132 | (98.51) | 211 | (99.53) | 70 | (97.22) | — |
| Yes | 6 | (0.88) | 1 | (0.38) | 2 | (1.49) | 1 | (0.47) | 2 | (2.78) |  |
| Respiratory | 3 | (0.44) | 0 | (0) | 1 | (0.75) | 1 | (0.47) | 1 | (1.39) |  |
| Thyroid | 1 | (0.15) | 0 | (0) | 0 | (0) | 0 | (0) | 1 | (1.39) |  |
| Nervous | 1 | (0.15) | 0 | (0) | 1 | (0.75) | 0 | (0) | 0 | (0) |  |
| Cardiovascular | 1 | (0.15) | 1 | (0.38) | 0 | (0) | 0 | (0) | 0 | (0) |  |
| Symptom, n(%) | Total (n=534) 4-18y(n=317) | | 0-3y (n=218) | | 4-6y (n=100) | | 7-14y (n=167) | | 15-18y (n=50) | |  |
| Fever (T≥37.3℃) | 341 | (63.86) | 155 | (71.1) | 57 | (57) | 99 | (59.28) | 30 | (60) | **0.032** |
| T_max_ (℃) | 39.0(38.55-39.5) | | 39.2(38.7-39.6) | | 39.1(38.5-39.4) | | 39.0(38.5-39.4) | | 39.1(38.6-39.4) | | 0.171 |
| Fever Duration (day) | 2 | (1-3) | 2 | (1-3) | 2 | (1-3) | 2 | (1-3) | 2 | (1-3) | 0.626 |
| Cough | 345 | (64.61) | 144 | (66.06) | 60 | (60) | 107 | (64.07) | 34 | (68) | 0.707 |
| Expectoration | 183 | (34.27) | 73 | (33.49) | 25 | (25) | 61 | (36.53) | 24 | (48) | **0.038*** |
| Throat discomfort | 86 | (27.31)^#^ | - | - | 22 | (22) | 42 | (25.15) | 22 | (44) | **0.012*** |
| Headache | 38 | (11.99)^#^ | - | - | 2 | (2) | 24 | (14.37) | 12 | (24) | **<0.001*** |
| Muscle or joint pain | 15 | (4.73)^#^ | - | - | 3 | (3) | 4 | (2.4) | 8 | (16) | **0.001*** |
| Tiredness | 38 | (11.99)^#^ | - | - | 6 | (6) | 21 | (12.57) | 11 | (22) | **0.016*** |
| Hyposmia | 2 | (0.37) | 2 | (0.63) | 0 | (0) | 0 | (0) | 2 | (4) | — |
| Diarrhea | 20 | (3.75) | 16 | (7.34) | 1 | (1) | 2 | (1.2) | 1 | (2) | **0.005** |

Note: Data are presented as case number (percentage %) or median (P_25_-P_75_). ★ All vaccinated patients had received the inactivated vaccine. P values were determined using the Chi-square test or Fisher’s exact test for categorical variables and Kruskal-Wallis H test for continuous variables. *Represent contrast of three groups (4-6y, 7-14y, 15-18y), and the others were a contrast of all four groups (0-3y, 4-6y, 7-14y, 15-18y). *#* Represent case number (percentage %) of 4-18y.

Supplementary Table 2. Laboratory results of all patients on admission

| **Laboratory results** | **0-3y (n=155)** | | **4-6y (n=88)** | | **7-14y (n=179)** | **15-18y (n=66)** | |  |
| --- | --- | --- | --- | --- | --- | --- | --- | --- |
| Leukocytes, 10^9^/L | 6.17(6.17-6.45) | 6.17(5.84-7.44) | | 5.2(4.09-6.17) ***### | | | 5.22(3.96-6.20) ***###$ | |
| Neutrophils, 10^9^/L | 3.89(2.65-3.89) | 3.89(2.58-4.58) * | | 2.98(2.08-3.89) *## | | | 3.30(1.86-4.43) | |
| Neutrophils (%) | 63.0(39.15-63.0) | 63(45.23-63.0) | | 61.60(47.7-67.45) * | | | 62.85(50.23-71.63) **# | |
| Lymphocytes, 10^9^/L | 1.59(1.59-2.69) | 1.59(1.59-2.67) | | 1.49(1.00-1.73) ***### | | | 1.20(0.86-1.59) ***###$$ | |
| Lymphocytes (%) | 25.8(25.80-45.30) | 25.85(25.80-39.13) | | 25.80(19.35-38.00) ** | | | 24.10(14.55-34.80) ***## | |
| Monocytes, 10^9^/L | 0.64(0.64-0.64) | 0.64(0.50-0.66) * | | 0.55(0.41-0.64) ***# | | | 0.60(0.42-0.73) ** | |
| Monocytes (%) | 10.4(10.40-10.40) | 10.40(8.28-10.40) ** | | 10.40(8.80-12.78) ## | | | 11.55(9.58-13.98) **###$ | |
| Basophils, 10^9^/L | 0.01(0.01-0.01) | 0.01(0.01-0.02) ** | | 0.01(0.01-0.02) *** | | | 0.01(0.01-0.02) | |
| Basophils (%) | 0.20(0.20-0.20) | 0.20(0.20-0.30) ** | | 0.30(0.20-0.40) *** | | | 0.20(0.20-0.40) * | |
| Eosinophils, 10^9^/L | 0.04(0.03-0.04) | 0.04(0.04-0.15) *** | | 0.04(0.01-0.11) # | | | 0.015(0-0.04) ***###$$$ | |
| Eosinophils (%) | 0.60(0.40-0.60) | 0.60(0.60-2.25) *** | | 0.60(0.25-2.20) ** | | | 0.30(0.10-1.00) *###$$$ | |
| Hemoglobin, g/L | 126(123-126) | 126(126-136) *** | | 136(126-142) ***### | | | 137(128.25-154) ***### | |
| Platelet, 10^9^/L | 240(234-240) | 240(240-285) ** | | 240(206.5-265.75) ## | | | 194.5(161-230.5) ***###$$$ | |

Note: Data are presented as median (P_25_-P_75_). N is the total number of patients with available data. P values were calculated by Mann–Whitney U-test. * Represent significant differences compared with 0-3y group; # represent significant differences compared with 4-6y group. $ represent significant differences compared with 7-14y group. */#/$ p<0.05, **/##/$$ p<0.01, ***/###/$$$ p<0.001.
